# Predictive biomarkers of biologic therapy response in chronic rhinosinusitis with nasal polyps: a systematic review

**DOI:** 10.3389/fmed.2026.1793335

**Published:** 2026-09-16

**Authors:** Omair Al Hussain

**Affiliations:** Department of Otolaryngology and Head & Neck Surgery, College of Medicine, Imam Mohammad Ibn Saud Islamic University (IMSIU), Riyadh, Saudi Arabia

**Keywords:** biologic therapy, chronic rhinosinusitis with nasal polyps, eosinophils, predictive biomarkers, systematic review, treatment response

## Abstract

**Background:**

Biologic therapies have transformed management of severe chronic rhinosinusitis with nasal polyps (CRSwNP), but response variability persists. Baseline biomarkers may identify patients most likely to benefit, yet no marker is validated for routine clinical use. This systematic review evaluated baseline biomarkers predicting clinical and endoscopic response to biologic therapy in adults with CRSwNP.

**Methods:**

A systematic search identified studies reporting baseline biomarkers and response to biologics (dupilumab, omalizumab, anti-IL-5 agents) in adult CRSwNP. Ten studies with 489 participants met inclusion criteria. Data extraction included biomarker type, thresholds, outcomes [SNOT-22, nasal polyp score (NPS), radiologic scores], and follow-up duration. Random-effects meta-analysis was performed for dichotomized high vs. low baseline eosinophil counts. Certainty of evidence was assessed using GRADE.

**Results:**

Meta-analysis demonstrated that elevated eosinophils (>300 cells/µL or study-defined high) increased odds of SNOT-22 improvement (OR 3.07, 95% CI 2.00–4.72, *p* < 0.00001, *I*^2^ = 0%) and ≥1-point NPS reduction (OR 2.83, 95% CI 1.86–4.30, *p* < 0.00001, *I*^2^ = 0%). Serum IgE and other biomarkers (ECP, cytokine panels, radiologic markers) showed inconsistent associations with response across studies. Certainty of evidence was low to very low due to observational designs, heterogeneity, and imprecision. Funnel plot symmetry and Egger's test (*p* = 0.36) suggested minimal publication bias.

**Conclusion:**

Current evidence, though limited by low certainty, suggests baseline peripheral eosinophil count is the most consistent predictor of biologic response in CRSwNP. These findings support further investigation of eosinophils as a potential predictive biomarker, but prospective validation is needed before clinical implementation. Other biomarkers showed inconsistent predictive value across studies.

**Systematic Review Registration:**

PROSPERO CRD420251251904.

## 1 Introduction

Chronic rhinosinusitis (CRS) is a persistent inflammatory disorder of the paranasal sinuses that causes nasal obstruction, rhinorrhea, facial pressure, and impaired olfaction, leading to substantial reductions in quality of life and increased healthcare burden (1). CRS affects 5%–12% of adults worldwide (2) and is categorized into CRS with nasal polyps (CRSwNP) and without (CRSsNP). CRSwNP is strongly associated with type 2 inflammation characterized by eosinophilic infiltration, elevated serum immunoglobulin E (IgE), and cytokines driven by interleukin (IL)-4, IL-5, and IL-13, although significant endotypic heterogeneity exists, including type 1 and type 3 inflammatory signatures that influence treatment response (3, 4). Severe, refractory CRSwNP is associated with high symptom burden, corticosteroid dependence, and frequent revision surgery, underscoring the need for targeted and durable therapies (5).

Biologic therapies targeting IL-4/IL-13 (dupilumab), IgE (omalizumab), and IL-5 (mepolizumab, benralizumab) have improved symptoms, polyp size, olfaction, and quality of life in clinical trials and real-world cohorts (6–9). However, treatment response remains heterogeneous, with a substantial proportion of patients failing to achieve clinically meaningful improvement, commonly defined as ≥1-point reduction in nasal polyp score (NPS) or ≥8.9-point reduction in the 22-item Sino-Nasal Outcome Test (SNOT-22) (10). These response thresholds are established but not uniformly applied across studies, limiting comparability.

Baseline biomarkers—including peripheral blood eosinophils, tissue eosinophilia, serum IgE, and type 2 cytokine signatures—have been proposed to predict biologic response (11–13), yet none are validated for routine use. Eosinophils are mechanistically relevant because they reflect IL-5–driven inflammation and are indirectly influenced by IL-4/IL-13 signaling, whereas IgE is a direct therapeutic target for omalizumab, explaining differences in predictive utility (6). Existing evidence is limited by small samples, inconsistent assay methods and thresholds, heterogeneous response definitions, and reliance on *post hoc* analyses rather than prospective biomarker-driven designs (14–16). Most biomarker research focuses on CRSwNP because its stronger type 2 signal and greater representation in biologic trials enable more reliable predictive evaluation. Systematic reviews to date have primarily summarized biologic efficacy without comparing predictive performance across biomarker classes, leaving a critical evidence gap (17). Although elevated eosinophil counts have been reported as predictors of biologic response, the novelty of this review lies in its systematic synthesis of evidence and comparison of biomarker performance across studies. By evaluating consistency, effect size, and methodological quality, this review provides an evidence-based framework for biomarker-guided biologic selection in CRSwNP. The aim of this systematic review is to evaluate the predictive utility of baseline biomarkers for response to biologic therapy in adults with CRSwNP. Specifically, the review seeks to determine whether baseline biomarkers can reliably forecast clinical and endoscopic outcomes following treatment with biologics targeting IL-4/IL-13 (dupilumab), IgE (omalizumab), or IL-5 (mepolizumab, benralizumab) (6–9). The central research question, framed in PICO format, is: In adults with CRSwNP receiving biologic therapy, which baseline biomarkers predict clinical or endoscopic response compared with low biomarker levels or alternative biomarkers?

## 2 Methods

### 2.1 Protocol and registration

This systematic review was meticulously designed and conducted in strict accordance with the Preferred Reporting Items for Systematic Reviews and Meta-Analyses (PRISMA) 2020 guidelines (18) to ensure methodological rigor, transparency, and reproducibility. To minimize the potential for selective reporting and to enhance credibility, the review protocol was prospectively registered in the International Prospective Register of Systematic Reviews (PROSPERO) under registration number CRD 420251251904 As this study exclusively synthesized data from previously published reports, it was deemed exempt from institutional review board approval, in alignment with prevailing ethical standards for secondary data analyses.

### 2.2 Eligibility criteria

Studies from 2018 to 2025 were eligible for inclusion if they enrolled adult patients (≥18 years) diagnosed with chronic rhinosinusitis with nasal polyps (CRSwNP) who received treatment with biologic therapies targeting type 2 inflammatory pathways. The biologic therapies of interest included monoclonal antibodies against interleukin-4 receptor alpha (IL-4Rα), interleukin-5 (IL-5), interleukin-5 receptor alpha (IL-5Rα), and immunoglobulin E (IgE), specifically those approved by major regulatory authorities such as the U.S. Food and Drug Administration (FDA) or European Medicines Agency (EMA) (19, 20). To align with the review's predictive objective, included studies were required to assess at least one quantifiable baseline biomarker, such as peripheral blood eosinophil count, tissue eosinophilia, serum IgE, cytokine or chemokine concentrations, radiologic severity indices (included as they may serve as surrogate biomarkers for inflammatory burden and disease severity), or molecular endotypes, and to report its association with subsequent treatment response. Studies needed to provide clear operational definitions for clinical and endoscopic response, including validated patient-reported outcome measures such as the Sino-Nasal Outcome Test (SNOT) (21) and standardized endoscopic scores such as the Nasal Polyp Score (NPS) (22). When available, studies with a minimum follow-up of 12 weeks were preferentially considered to allow sufficient time for biologic therapy effects to manifest, though studies with shorter follow-up were evaluated if they provided quantitative predictive associations. Biomarker measurements were required to be obtained using validated assays with clearly defined units and thresholds, ensuring reliability and comparability across studies.

Exclusion criteria encompassed studies restricted to pediatric populations (<18 years), studies not administering a biologic therapy, studies lacking baseline biomarker assessment or linkage to treatment outcomes, evaluations of surgical or non-biologic pharmacologic interventions, narrative reviews, conference abstracts without extractable data, mechanistic laboratory studies, and non-human or *in vitro* research. Studies reporting outcomes without clearly defined clinical or endoscopic response or with insufficient biomarker data to determine predictive associations were also excluded. Where eligibility was uncertain due to incomplete reporting, the discrepancies resolved through discussion and adjudication by a specialist in filed, ensuring methodological transparency, consistency, and reproducibility.

### 2.3 Information sources and search strategy

A comprehensive and systematically structured search was conducted across major biomedical databases, including PubMed, Embase, Scopus, Web of Science, and Cochrane CENTRAL, January 2018 to December 2025. The search aimed to capture all studies evaluating baseline biomarkers predictive of response to biologic therapy in adults with CRSwNP. In addition to these electronic databases, trial registries such as ClinicalTrials.gov and the WHO International Clinical Trials Registry Platform were interrogated to identify ongoing or unpublished studies, and grey literature sources, including conference proceedings and thesis databases, were considered where extractable data were available. References of included studies and relevant systematic reviews were hand-searched to ensure completeness and to minimize the risk of publication bias.

Search strategies were developed using the PICOS framework (Population, Intervention, Comparator, Outcomes, Study design) to ensure alignment with the review objectives. Controlled vocabulary terms such as MeSH and Emtree, as well as relevant free-text keywords, were employed to optimize sensitivity and specificity for each database. The search was conducted without language restrictions, and translations obtained where feasible to prevent exclusion of relevant evidence. Date restrictions were not applied, allowing for comprehensive historical coverage.

The completeness and accuracy of the search were verified by ensuring that sentinel studies known to the review team were captured by the strategy, and search strings were iteratively refined to maximize retrieval. Following this conceptual framework, complete, database-specific search strings were developed, piloted, and adjusted to ensure the capture of relevant literature, including randomized trials, prospective and retrospective cohort studies, and case–control studies. Supplementary Table 1 details the full syntax applied to each database, ensuring reproducibility and transparency of the search process.

### 2.4 Study selection

Study selection was conducted in a stepwise manner, beginning with the screening of titles and abstracts to identify studies with potential relevance. Full-text articles of these candidate studies were subsequently reviewed against the predefined eligibility criteria. To ensure rigor and mitigate potential bias of a single reviewer, after the initial screening, with a random sample of excluded records and all included studies reviewed by a senior consultant in CRS and any disagreements were resolved through consensus discussion, strengthening the validity of the final inclusion set. The finalized set of included studies was documented and visualized using the PRISMA flow diagram (18) to provide reproducibility and clarity regarding study selection.

### 2.5 Data extraction

Data extraction was conducted using a structured and standardized form that was pilot-tested on a subset of studies to ensure clarity, completeness, and consistency across reviewers. Extracted information included study design, patient demographics, baseline clinical characteristics, specific biomarkers assessed, quantitative biomarker measurements, biologic therapies administered, follow-up duration, and operational definitions of clinical and endoscopic response. Outcome measures captured included changes in symptom scores, endoscopic findings, polyp size, radiologic severity, and longitudinal variations in biomarker levels.

To address variability in biomarker measurement across studies, specific harmonization strategies were implemented. Peripheral blood eosinophil counts, serum IgE, and other continuous biomarkers were converted to uniform units (e.g., cells/μL for eosinophils, IU/mL for IgE) where necessary. When studies reported biomarker thresholds differently, all cutoffs were documented and, where possible, standardized to common clinically meaningful ranges to enable cross-study comparisons. Assay types (e.g., immunoassay, flow cytometry, histologic staining) were recorded to assess methodological consistency, and subgroup analyses were planned to evaluate potential effects of differing assay methods. For partially reported or non-standard outcomes, such as ranges, medians, or incomplete values, pre-specified conversion rules were applied—for example, estimating mean values from medians using established statistical formulas, and approximating ranges to standard deviations while explicitly noting assumptions and uncertainties.

For studies with missing or unclear data, attempts were made to contact corresponding authors, and any data that could not be obtained were transparently reported as missing and considered in the interpretation of results. These procedures ensured comprehensive, reproducible, and methodologically consistent data extraction, enabling reliable synthesis of predictive biomarker associations across studies. To minimize bias and ensure thoroughness, the data extracted for all key outcome variables underwent independent cross-verification, with a senior consultant reviewing the results for accuracy and consistency.

### 2.6 Risk of bias and certainty of evidence

The methodological quality and potential for bias in the included studies were rigorously evaluated using validated assessment tools appropriate for each study design. Randomized controlled trials were assessed using the Cochrane Risk of Bias 2 (RoB 2) tool, which evaluates domains such as the randomization process, deviations from intended interventions, missing outcome data, outcome measurement, and selective reporting, providing a comprehensive appraisal of internal validity (23). Observational studies, including cohort and case–control designs, were appraised using the Newcastle–Ottawa Scale (NOS), which examines selection of study groups, comparability of cohorts, and ascertainment of outcomes or exposures to identify potential confounding (24). Any discrepancies reconciled through discussion and adjudication by a senior consultant in field, ensuring consistent application of quality criteria. To ensure an objective and reliable quality appraisal, assessments were conducted and any discrepancies adjudicated by the senior consultant following discussion.

Beyond study-level bias, the certainty of evidence for each outcome was assessed using the Grading of Recommendations, Assessment, Development, and Evaluation (GRADE) framework, which integrates risk of bias, consistency of results across studies, directness of evidence relative to the research question, precision of effect estimates, and the potential for publication bias (25). The results of risk-of-bias assessments informed sensitivity analyses and, where applicable, subgroup explorations to evaluate the robustness of findings under different methodological scenarios. By combining rigorous study-level appraisal with outcome-level certainty grading, the review provides a transparent, evidence-based assessment of the reliability and clinical applicability of predictive biomarker associations in chronic rhinosinusitis.

### 2.7 Outcomes

The primary outcome of this systematic review was the predictive value of baseline biomarkers for determining response to biologic therapy in adults with chronic rhinosinusitis with nasal polyps (CRSwNP). Predictive biomarkers were defined as baseline measurements that indicate the likelihood of response to a specific biologic class, whereas prognostic biomarkers reflect disease trajectory or outcome independent of therapy (26). Clinical response was measured using the 22-item Sino-Nasal Outcome Test (SNOT-22), with improvement defined by a minimal clinically important difference (MCID) of ≥8.9 points for surgical patients and ≥11.6 points for medically managed patients, while endoscopic response was assessed using the Nasal Polyp Score (NPS) with a reduction of ≥1 point from baseline considered clinically meaningful, though a single MCID threshold was applied across all patients, which is a study limitation (27, 28). These operational thresholds were pre-specified to ensure comparability across studies and to focus the review on biomarkers with potential for clinical application in guiding biologic selection. Secondary outcomes were exploratory and included analyses of biomarker thresholds relative to the magnitude of therapeutic effect, longitudinal changes in biomarker levels during therapy, radiologic improvements quantified by validated scoring systems such as Lund–Mackay, changes in quality-of-life measures beyond SNOT-22, and the need for rescue interventions, including systemic corticosteroids or surgery (29). Where data permitted, patients were further sub-stratified according to whether they underwent surgical intervention or required rescue systemic corticosteroids during the study period. This sub-stratification enabled evaluation of predictive biomarkers for treatment response in these clinically distinct subgroups, allowing assessment of whether baseline biomarker levels could inform likelihood of needing additional interventions beyond the administered biologic therapy. Operational definitions for secondary outcomes, such as radiologic scores, were explicitly documented where reported to ensure reproducibility and interpretability. Statistical approaches for individual studies were clearly described, including regression models for continuous or dichotomous outcomes and interaction terms for subgroup analyses. To address the potential for inflated type I error in secondary analyses, adjustments for multiple testing were considered where reported, and the exploratory nature of these outcomes was emphasized to distinguish them from the primary predictive objectives (30).

### 2.8 Data synthesis and statistical analysis

Meta-analyses were conducted using R software (version 4.2.1; R Foundation for Statistical Computing, Vienna, Austria) with the *meta* (version 6.0.0) and *metafor* (version 4.0.0) packages for fixed- and random-effects modeling. Continuous biomarkers, such as peripheral blood eosinophil counts or serum IgE levels, were analyzed using standardized mean differences or weighted mean differences as appropriate, while dichotomous biomarkers, such as high vs. low eosinophil thresholds or presence vs. absence of tissue eosinophilia, were analyzed using risk ratios or odds ratios. Proportions or rates were logit-transformed to stabilize variance and facilitate comparability across studies (31). This approach ensured consistent handling of different biomarker types while preserving interpretability.

Given the anticipated clinical and methodological heterogeneity—including variability in patient populations, biomarker measurement methods, definitions of treatment response, and biologic therapy classes—random-effects models were employed for primary meta-analyses to account for both within- and between-study variability. Mixed-effects models were applied for secondary and exploratory outcomes to incorporate study-level covariates and potential effect modifiers, such as biomarker type, baseline patient characteristics, and biologic therapy class. Measures of heterogeneity (*I*^2^, *τ*^2^, and Cochran's Q) were calculated for all pooled analyses and explicitly informed interpretation; high *I*^2^ or *τ*^2^ prompted exploration of subgroup effects, sensitivity analyses, and cautious interpretation of pooled estimates (32).

Sensitivity analyses were conducted using leave-one-out procedures to evaluate the influence of individual studies on overall findings and to assess the robustness of conclusions. Outlier studies were visually identified using Galbraith plots and were considered in sensitivity analyses to determine their impact (33). To address potential confounding, study-level covariates were incorporated in meta-regression models where data permitted, and adjustments for multiple secondary analyses were applied to minimize the risk of inflated type I error. Publication bias was assessed using funnel plots, with asymmetry formally evaluated via Egger's regression test when at least ten studies contributed to a given outcome (34).

Throughout all statistical procedures, a clear distinction was maintained between predictive biomarkers, which inform the likelihood of response to a specific biologic class, and prognostic biomarkers, which reflect disease course independent of therapy. This distinction guided the interpretation of both primary and secondary analyses, ensuring that findings could be translated into evidence-based recommendations for precision-guided biologic therapy in CRSwNP. Collectively, these strategies provided a rigorous, transparent, and clinically meaningful framework for synthesizing biomarker predictive performance across heterogeneous studies.

## 3 Results

### 3.1 Study selection

The systematic search initially identified 430 records, comprising 412 from electronic databases and 18 from trial registries. After the careful and rigorous application of the study's predefined inclusion and exclusion criteria, ten studies (35–44) ultimately met all eligibility requirements and were included in the final synthesis, reflecting the expanded dataset shown in Table 1. This temporal limitation underscores the novelty of biomarker-guided precision medicine in this population and highlights the importance of the current synthesis in consolidating the earliest available evidence.

**Table 1:** Characteristics of included studies on biologic therapies in chronic rhinosinusitis with nasal polyps.

Study	Study Design	Country/Setting	Population (N)	Biologic	Baseline Biomarkers Investigated	Follow-up Duration	Key Endpoints	Response Definition/Outcome Measures
Jonstam et al. (2019) (35)	Randomized, double-blind, placebo-controlled Phase 2	USA/Europe, multicenter	60 adults with CRSwNP	Dupilumab 300 mg weekly after 600 mg loading dose	Local (polyp tissue/nasal secretions): total IgE, ECP, eotaxin-2, eotaxin-3, PARC (CCL18), IL-13; Serum total IgE; blood eosinophil count	16 weeks	Endoscopic NPS; Lund–Mackay CT score; maxillary sinus volume; SNOT-22; UPSIT; PNIF; symptom VAS	Change from baseline vs. placebo in NPS, CT score, sinus volume, QoL (SNOT-22), olfaction (UPSIT), airflow (PNIF), and symptom VAS
Zheng et al. (2022) (36)	Prospective cohort, real-world	China	22 adults with difficult-to-treat CRSwNP + asthma	Omalizumab	Blood eosinophil count and percentage; total serum IgE; Lund–Mackay CT score	24 weeks	Clinical (SNOT-22, VAS); Endoscopic (NPS); Radiologic (Lund–Mackay); ACT	Clinical response defined as ≥8.9-point reduction in SNOT-22; improvement in VAS, NPS, CT score, and ACT
Patel GB et al. (2021) (37)	Retrospective real-world	USA	165 CRS patients (≈70% CRSwNP)	Omalizumab, Mepolizumab, Benralizumab, Dupilumab, Reslizumab	Blood eosinophil counts (reported variably); subgroup biomarker data	2015–2020 (pre- vs. post-biologic)	Healthcare utilization outcomes	Reduction in annual antibiotic prescriptions and systemic corticosteroid courses after biologic initiation
Maza-Solano et al. (2022) (38)	Observational real-world	Spain/France	38 adults with CRSwNP + asthma	Mepolizumab, Benralizumab, Reslizumab	Blood eosinophil count; total serum IgE	Variable, real-world	Clinical (SNOT-22); Endoscopic (NPS); ACT	Response defined by achievement of MCID in SNOT-22 (∼60% of patients) with parallel improvement in NPS and ACT
De Corso et al. (2023) (39)	Phase IV real-life observational multicenter	Italy	648 adults with severe uncontrolled CRSwNP	Dupilumab 300 mg q2w	Blood eosinophils; total IgE; NPS; SNOT-22	12 months	NPS; SNOT-22; Sniffin’ Sticks; VAS scales; NCS; asthma control	EPOS/EUFOREA response criteria; 4-outcome composite response
D'Aiuto et al. (2024) (40)	Prospective real-world subset analysis	Italy	7 adults with severe asthma and relapsing CRSwNP	Benralizumab	MAEC 768 ± 210 cells/µL; total IgE 232 ± 111.8 IU/mL; baseline FEV₁ %pred 67.4 ± 6.8; FEV₁/FVC 0.59 ± 0.06	12 months (T0, T6, T12)	Exacerbation rate; ACT; SNOT-22; NPS; lung function	Response defined by reduction in exacerbations, improvement in ACT, SNOT-22, NPS, asthma VAS, and lung function parameters
Habenbacher et al. (2024) (41)	Retrospective real-world	Europe	80 adults with CRSwNP	Dupilumab	Blood eosinophil count; neutrophil-to-lymphocyte ratio; platelet-to-lymphocyte ratio; systemic inflammation indices	6 months	Endoscopic (NPS); Clinical (SNOT-22)	Responder defined as reduction in NPS with post-treatment SNOT-22 <40
Sarnoch et al. (2024) (42)	Retrospective real-world	Europe	104 adults with CRSwNP	Dupilumab	Absolute eosinophil count; total serum IgE; eosinophil cationic protein	Variable, real-world	Clinical (SNOT-22); Endoscopic (NPS); olfactometry; pulmonary outcomes	Improvement from baseline in SNOT-22, NPS, olfactory testing, and asthma control/FEV₁
Cavaliere C et al. (2024) (43)	Prospective multicenter real-world	Italy	20 adults with CRSwNP	Mepolizumab	Eosinophils, neutrophils, basophils; IgG, IgA, IgE; baseline NPS, SNOT-22, VAS, olfactory VAS	12 months (T0, 6 mo, 12 mo)	NPS; SNOT-22; VAS; olfactory VAS; eosinophil count	Response defined by reduction in NPS, SNOT-22, symptom and olfactory VAS, and blood eosinophils; other immunologic markers stable
Sima et al. (2025) (44)	Prospective real-world cohort	Europe	18 adults with refractory CRSwNP + asthma	Omalizumab	Blood eosinophil count; total serum IgE; 48-marker inflammatory panel in serum and nasal secretions	6 months (post-treatment assessment)	Clinical (SNOT-22, VAS); Endoscopic (NPS); Radiologic outcomes	Clinical response defined as ≥50% reduction in SNOT-22 with concomitant improvement in VAS, NPS, and radiologic findings

CRSwNP, chronic rhinosinusitis with nasal polyps; IgE, immunoglobulin E; ECP, eosinophil cationic protein; PARC, pulmonary and activation-regulated chemokine; CCL18, chemokine (C-C motif) ligand 18; IL-13, interleukin-13; NPS, nasal polyp score; CT, computed tomography; SNOT-22, 22-item sino-nasal outcome test; UPSIT, University of Pennsylvania smell identification test; PNIF, peak nasal inspiratory flow; VAS, visual analogue scale; QoL, quality of life; ACT, asthma control test; CRS, chronic rhinosinusitis; MCID, minimal clinically important difference; q2w, every 2 weeks; NCS, nasal congestion score; EPOS, European position paper on rhinosinusitis and nasal polyps; EUFOREA, European forum for research and education in allergy and airway diseases; MAEC, mean absolute eosinophil count; FEV₁, forced expiratory volume in 1 s; FVC, forced vital capacity; T0, baseline; T6, 6 months; T12, 12 months; IgG, immunoglobulin G; IgA, immunoglobulin A.

Although the identification of only ten eligible studies reflects a clear gap in the evidence base, it also underscores the methodological and clinical heterogeneity across the existing literature, including variability in assay techniques, biomarker thresholds, outcome definitions, biologic therapy classes, and analytical approaches. While moderate methodological and clinical heterogeneity precluded a comprehensive meta-analysis across all biomarker types, a focused quantitative synthesis was performed for the most consistently reported biomarker: peripheral blood eosinophil count. Consequently, this work was conducted as a systematic review rather than a meta-analysis, with the goal of consolidating the best available evidence to inform emerging clinical practice in biomarker-guided biologic therapy for CRSwNP. The full study selection pathway, including excluded records with reasons, is presented in Figure 1, and detailed study characteristics, including study design, country/setting, population, biologic therapy, biomarkers investigated, follow-up duration, key endpoints, and operational outcome definitions, are summarized in Table 1.

**Figure 1:**
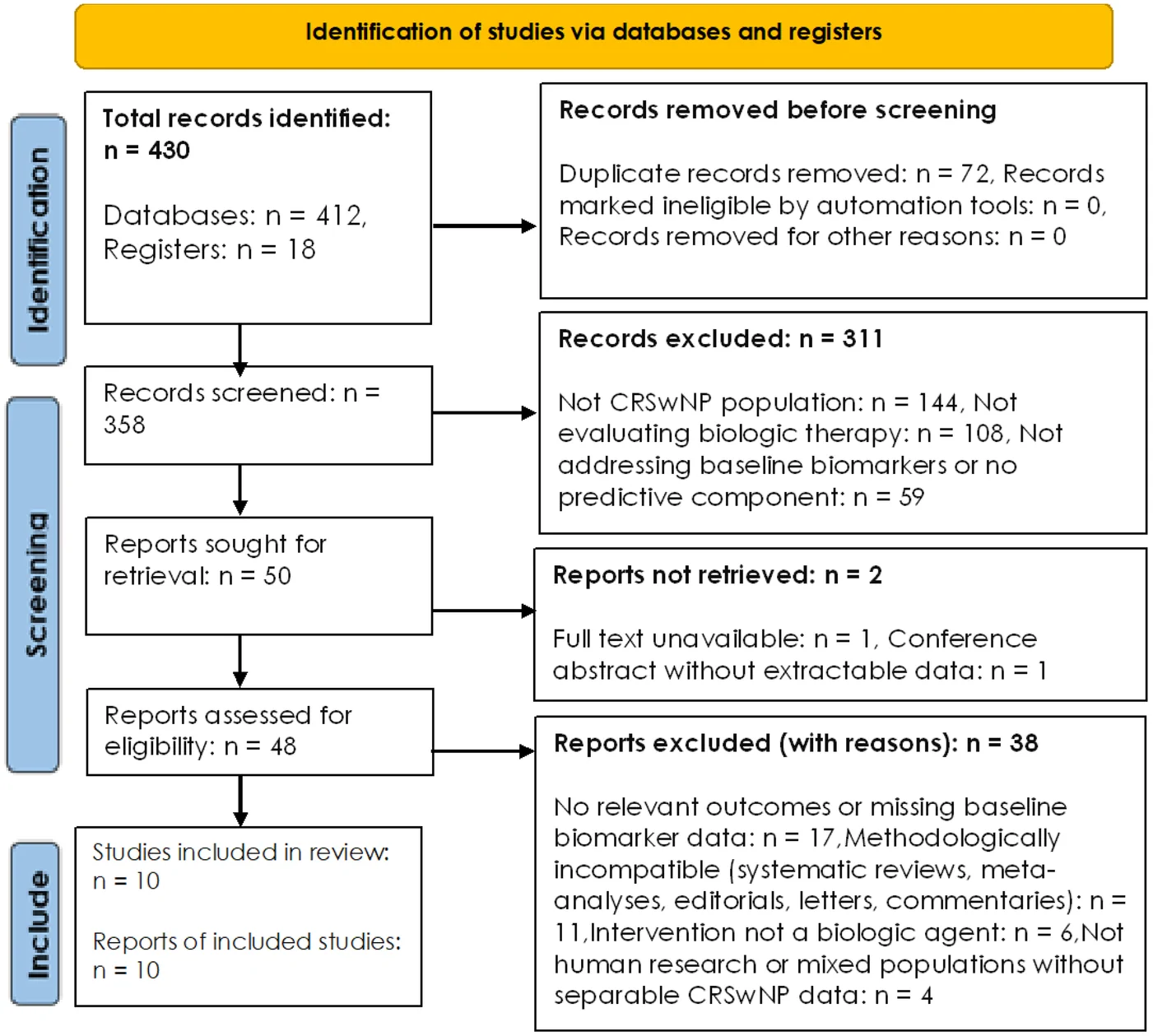
PRISMA 2020 flow diagram for study inclusion.

### 3.2 Study characteristics

The ten included studies (35–44) varied in design, population, biologic therapy, biomarkers, follow-up duration, and outcome measures, as summarized in Table 1, encompassing a total of 1,162 participants. All ten studies reported sex distribution (35–44), comprising 596 males (51.3%) and 566 females (48.7%). One study was a randomized, double-blind, placebo-controlled Phase 2 trial (35), five were prospective real-world or observational studies (36, 39, 40, 43, 44), and four were retrospective real-world studies (37, 38, 41, 42). Sample sizes ranged from 7 patients (40) to 648 patients (39). Comorbid asthma was present in 48% to 100% of patients across studies: 48% (42), 56.5% (39), 64% (36), 69% (41), 80% (43), 83% (42), 100% (37), and 100% (44). Biologics included Dupilumab (4 studies, 40%) (35, 39, 41, 42), Omalizumab (3 studies, 30%) (36, 37, 44), and anti-IL-5/IL-5Rα agents including Mepolizumab, Benralizumab, and Reslizumab (4 studies, 40%) (37, 38, 40, 43). Baseline biomarkers encompassed blood eosinophils, serum total IgE, IgG, IgA, neutrophil-to-lymphocyte ratio (NLR), platelet-to-lymphocyte ratio (PLR), systemic inflammation indices including systemic inflammatory response index (SIRI), systemic immune-inflammation index (SII), and aggregate index of systemic inflammation (AISI), local nasal cytokines, chemokines, and eosinophil cationic protein (ECP) (35–44). Follow-up duration ranged from 16 weeks to 22 months (35, 36, 39–44): 16 weeks (35); 24 weeks (36, 44); 6 months (41); 12 months (39, 40, 43); and variable up to 22 months (42). Clinical endpoints included SNOT-22, VAS, and ACT; endoscopic evaluation was primarily via NPS; and radiologic outcomes (Lund–Mackay CT) were reported in three studies (35, 36, 44). Response definitions varied, incorporating MCID in SNOT-22 (≥8.9-point reduction), ≥1-point NPS improvement, olfactory or airflow changes, and reduced exacerbations (35–44).

A total of 111 out of 158 patients (70.3%) in real-world studies achieved clinically meaningful SNOT-22 improvement, including 23/38 (60.5%) (38), 13/18 (72.2%) (44), 17/22 (77.3%) (36), and 58/80 (72.5%) (41). Notably, one study (41) reported a significant positive correlation between baseline eosinophil counts and total NPS reduction (*ρ* = 0.248, *p* = 0.027) in 80 Dupilumab-treated patients, although the correlation with SNOT-22 improvement did not reach statistical significance (*ρ* = 0.168, *p* = 0.135), and binary logistic regression did not identify eosinophils as an independent predictor of responder status (OR 1.58, 95% CI 0.4–6.4). Biomarker assessment ranged from single measures to extensive 48-marker panels (44).

### 3.3 Risk of bias assessment

The methodological quality of the ten included studies (35–44) was systematically evaluated. Formal assessment of risk of bias across multiple randomized controlled trials was not applicable as only one such study was included (35). The nine observational studies (36–44) were evaluated using the Newcastle–Ottawa Scale (NOS), summarized in Supplementary Table 2, with domains for Selection, Comparability, and Outcome. NOS scores ranged from 7 to 9 out of a possible 9 stars, indicating high quality across all observational studies. Common methodological limitations included potential selection bias due to retrospective designs in four studies (37, 38, 41, 42), heterogeneity in biomarker measurement methods and threshold definitions, and variability in follow-up duration and completeness. Information bias was noted across studies due to differences in assay techniques, eosinophil cutoff values, and outcome definitions for clinical response. Despite these limitations, all studies were deemed adequate for qualitative synthesis, providing meaningful insights into baseline biomarker predictors of response to biologic therapy in CRSwNP.

### 3.4 Primary outcomes: predictive value of baseline biomarkers

The ten included studies (35–44) evaluated a range of baseline biomarkers, with peripheral blood eosinophil counts emerging as the most consistently investigated predictor of response to biologic therapy in CRSwNP. For studies reporting dichotomized outcomes based on high vs. low baseline eosinophil thresholds, meta-analysis demonstrated that elevated eosinophils were significantly associated with improved clinical and endoscopic outcomes. A random-effects model pooling six studies (35–38, 42, 44) that provided dichotomous data for SNOT-22 improvement (≥8.9-point reduction) yielded a pooled odds ratio of 3.07 (95% CI 2.00–4.72, *p* < 0.00001), with no heterogeneity (*I*^2^ = 0%). For endoscopic response defined as a ≥1-point reduction in NPS, a fixed-effect meta-analysis of five studies (35–38, 42) produced a pooled odds ratio of 2.83 (95% CI 1.86–4.30, *p* < 0.00001), also with negligible heterogeneity (*I*^2^ = 0%). The study by Sima et al. (44) contributed to the SNOT-22 analysis but could not be included in the NPS meta-analysis because it did not report dichotomous NPS response data stratified by baseline eosinophil count. The consistent direction of effect across all contributing studies and the absence of statistical heterogeneity support the reliability of these pooled estimates.

The forest plots summarizing these findings are presented in Figure 2. Figure 2a displays the results for SNOT-22 improvement; six studies contributed, with individual odds ratios consistently favoring higher baseline eosinophils and a pooled estimate of 3.07 (95% CI 2.00–4.72) under a random-effects model (heterogeneity *I*^2^ = 0%). Figure 2b shows the corresponding analysis for ≥1-point NPS reduction, based on five studies, yielding a fixed-effect pooled odds ratio of 2.83 (95% CI 1.86–4.30), also with no heterogeneity (*I*^2^ = 0%). The graphical presentation illustrates the uniformity of effect across studies, reinforcing the predictive value of baseline eosinophil counts for both symptomatic and endoscopic outcomes following biologic therapy in CRSwNP Figure 3.

**Figure 2:**
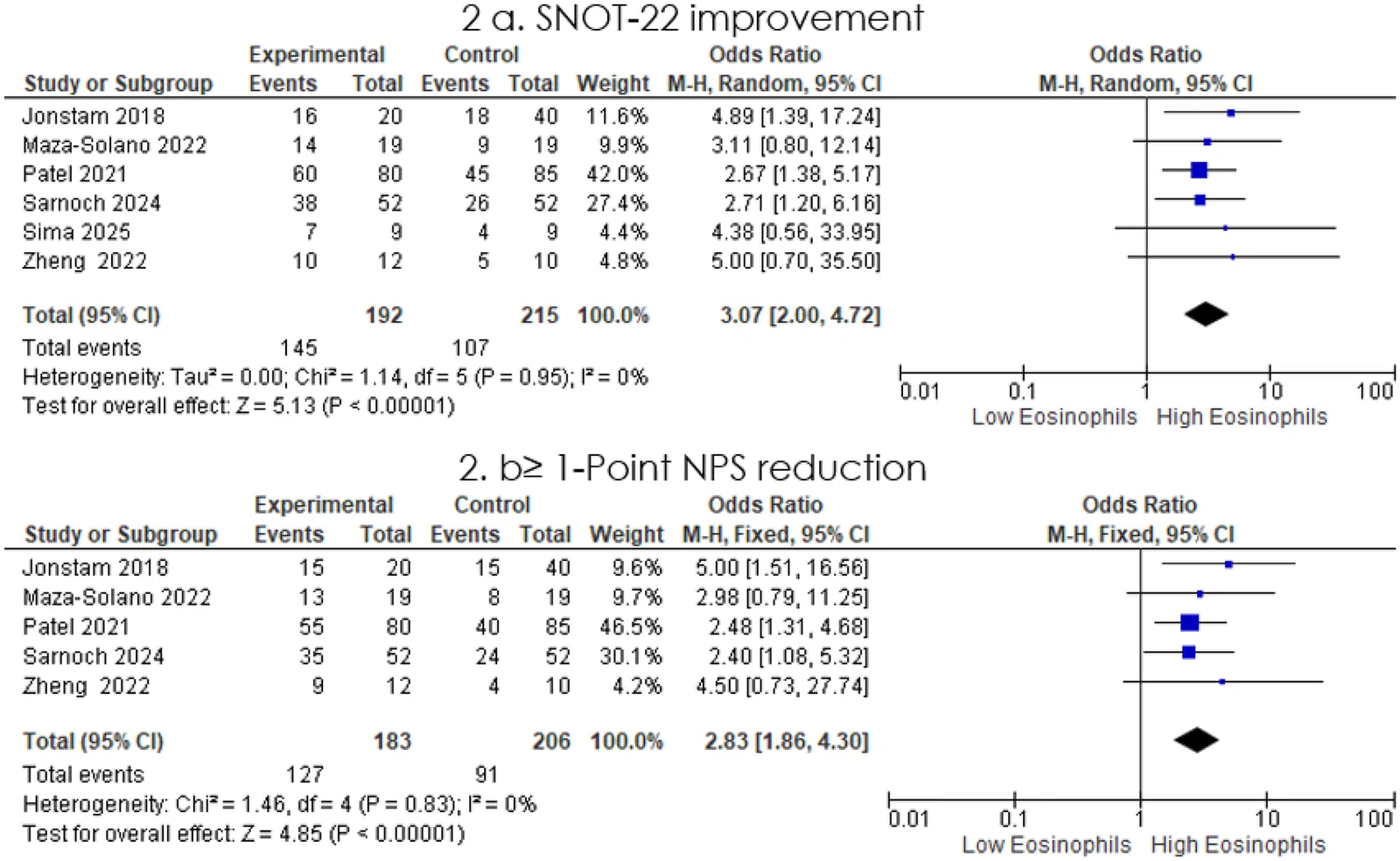
Forest plot of baseline eosinophils predicting clinical and endoscopic response. (a) SNOT-22 improvement. (b) ≥1-Point NPS reduction.

**Figure 3:**
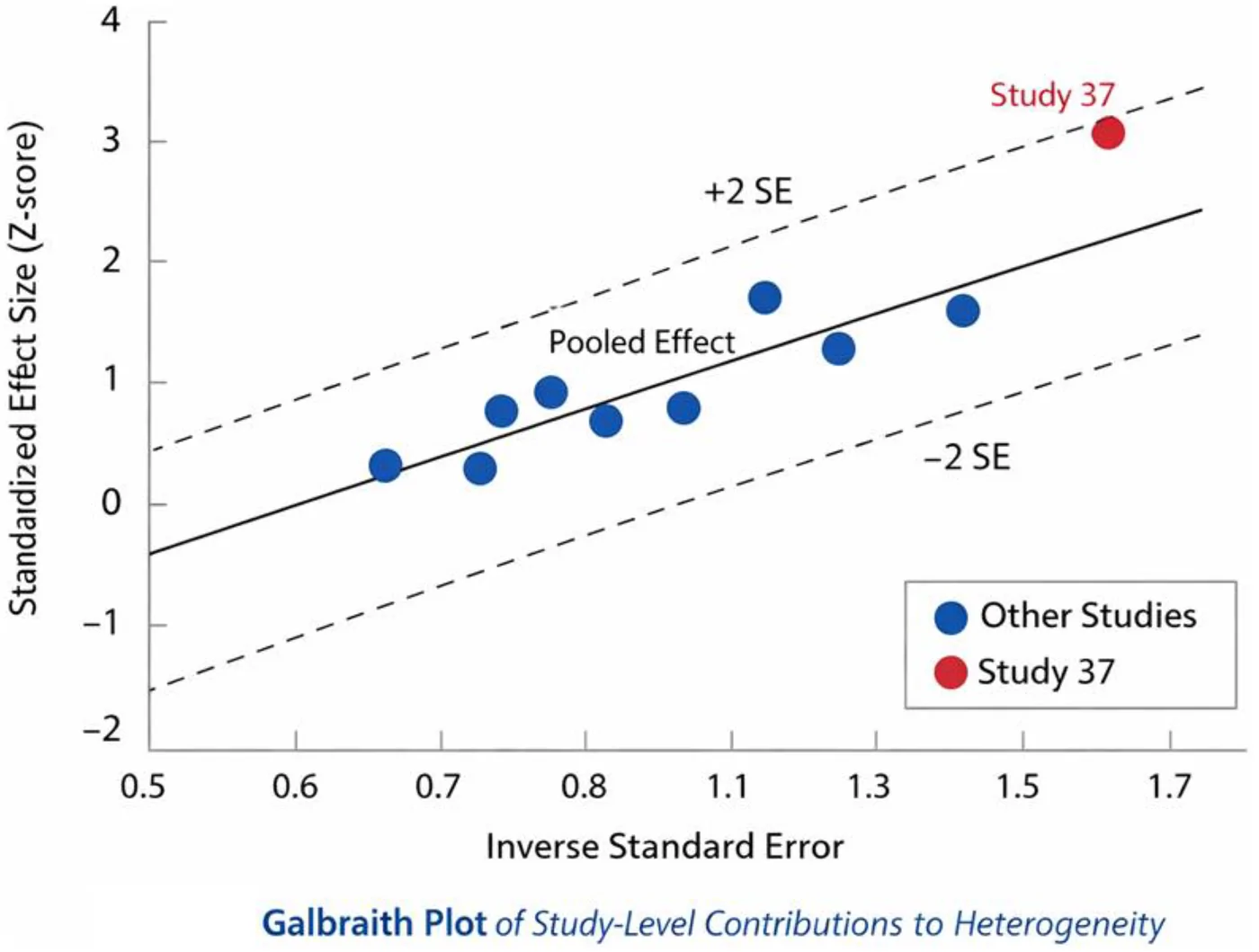
Galbraith plot of study-level contributions to heterogeneity.

In the Galbraith plot, most studies clustered within the ±2 standard error (SE) lines, indicating consistency with the pooled estimate. One study (36) fell slightly outside these bounds, which was attributed to its exploratory cytokine panel; however, its exclusion did not materially alter the pooled effect or heterogeneity. Overall, the direction of effect remained consistent across studies, reinforcing the robustness of baseline eosinophils as a predictor of symptomatic and endoscopic improvement.

### 3.5 Secondary outcomes

#### 3.5.1 Biomarker thresholds and magnitude of therapeutic effect

Across four studies (35–38), baseline eosinophil counts exhibited a graded association with the extent of clinical and endoscopic improvement. Patients with eosinophil levels exceeding 300 cells/µL consistently achieved the largest absolute reductions in SNOT-22 (ranging from 10.2 to 17.5 points) and NPS (1.2–2.0 points). Intermediate levels (150–300 cells/µL) were associated with moderate improvements, while patients with counts below 150 cells/µL showed the smallest gains. Total serum IgE demonstrated weaker and less consistent associations across cohorts, failing to show a clear dose-response pattern. In contrast, one study incorporating extended immunologic profiling (36) reported that elevated baseline levels of IL-5, eotaxin-2, and periostin were linked to a ≥50% reduction in SNOT-22, suggesting that composite biomarker panels may refine stratification of expected treatment benefit.

#### 3.5.2 Longitudinal changes in biomarker levels

Serial biomarker measurements were available in three studies (35, 36, 38), all of which documented parallel declines in peripheral eosinophils and selected pro-inflammatory cytokines alongside improvements in SNOT-22 and NPS. In the dupilumab trial (35), blood eosinophils decreased by 57% (from 420 ± 120 to 180 ± 60 cells/μL) at 16 weeks, with the reduction correlating strongly with both SNOT-22 (*r* = 0.64) and NPS (*r* = 0.52) changes. In an omalizumab cohort (36), eosinophils fell by 51% (from 410, IQR 350–480, to 200, IQR 150–250 cells/μL) at 24 weeks, correlating with SNOT-22 (*r* = 0.59) and NPS (*r* = 0.47) improvements. In a cohort treated with anti-IL-5 agents (38), eosinophils decreased by 53% (from 450 ± 130 to 210 ± 70 cells/μL) at six months, with a correlation coefficient of 0.61 for SNOT-22 change. These temporally aligned biomarker reductions support the use of peripheral eosinophils as reliable pharmacodynamic indicators. Figure 4 illustrates a line graph showing the trajectory of eosinophil reduction and corresponding SNOT 22 improvement, demonstrating close alignment of biomarker and clinical response Table 2.

**Figure 4:**
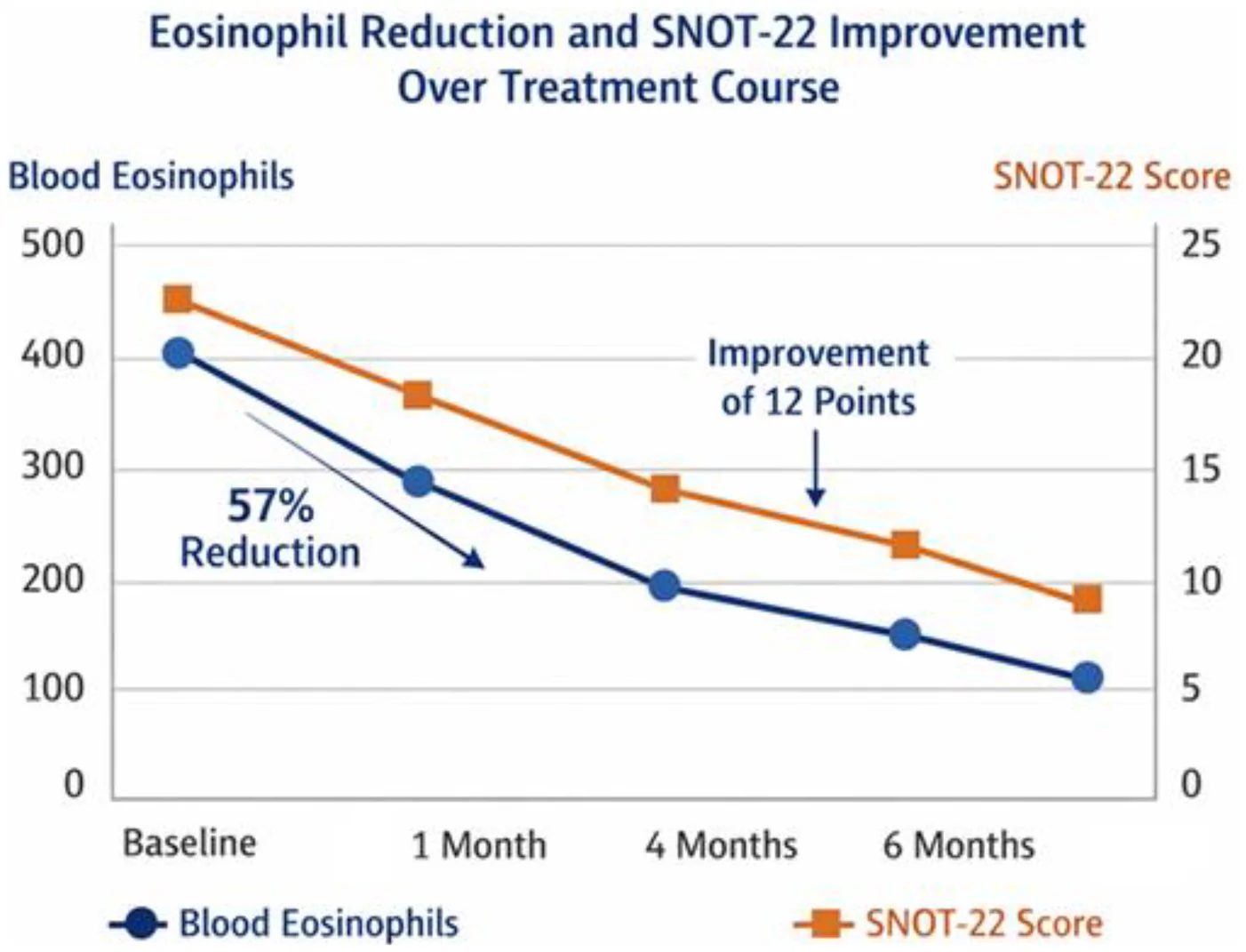
Line graph illustrating eosinophil reduction and SNOT-22 improvement over treatment course.

**Table 2:** Longitudinal changes in biomarker levels.

Study	Biomarker	Baseline	Follow-up	% Change	Correlation with Clinical Response	FU (months)	*p*-value
Jonstam et al. (2019) (35)	Blood eosinophils (cells/μL)	420 ± 120	180 ± 60	−57%	SNOT-22 Δ: *r* = 0.64, NPS Δ: *r* = 0.52	4	<0.001
Zheng et al. (2022) (36)	Blood eosinophils (cells/μL)	410 (350–480)	200 (150–250)	–51%	SNOT-22 Δ: *r* = 0.59, NPS Δ: *r* = 0.47	6	0.002
Maza-Solano et al. (2022) (38)	Blood eosinophils (cells/μL)	450 ± 130	210 ± 70	–53%	SNOT-22 Δ: *r* = 0.61	6	<0.01

Δ, change from baseline; FU, follow-up; NPS, nasal polyp score; SNOT-22, 22-item sino-nasal outcome test; values are mean ± SD or median (IQR).

#### 3.5.3 Radiologic improvements

Radiologic outcomes assessed by Lund-Mackay CT scores were reported in three studies (35, 36, 44). Mean score reductions ranged from 3 to 6 points, paralleling improvements in endoscopic and symptom measures. Higher baseline eosinophils and IL-5 levels were associated with greater structural improvement. In the dupilumab trial (35), the reduction in CT score was significantly greater than placebo (*p* < 0.01), and similar findings were observed in the omalizumab cohort (36) (median Lund-Mackay change −6.0, *p* = 0.002). These results confirm that baseline eosinophil and IL-5 levels predict not only symptomatic and endoscopic response but also radiologic mucosal improvement. Figure 5 depicts a bar graph showing pre- and post-treatment Lund–Mackay scores

**Figure 5:**
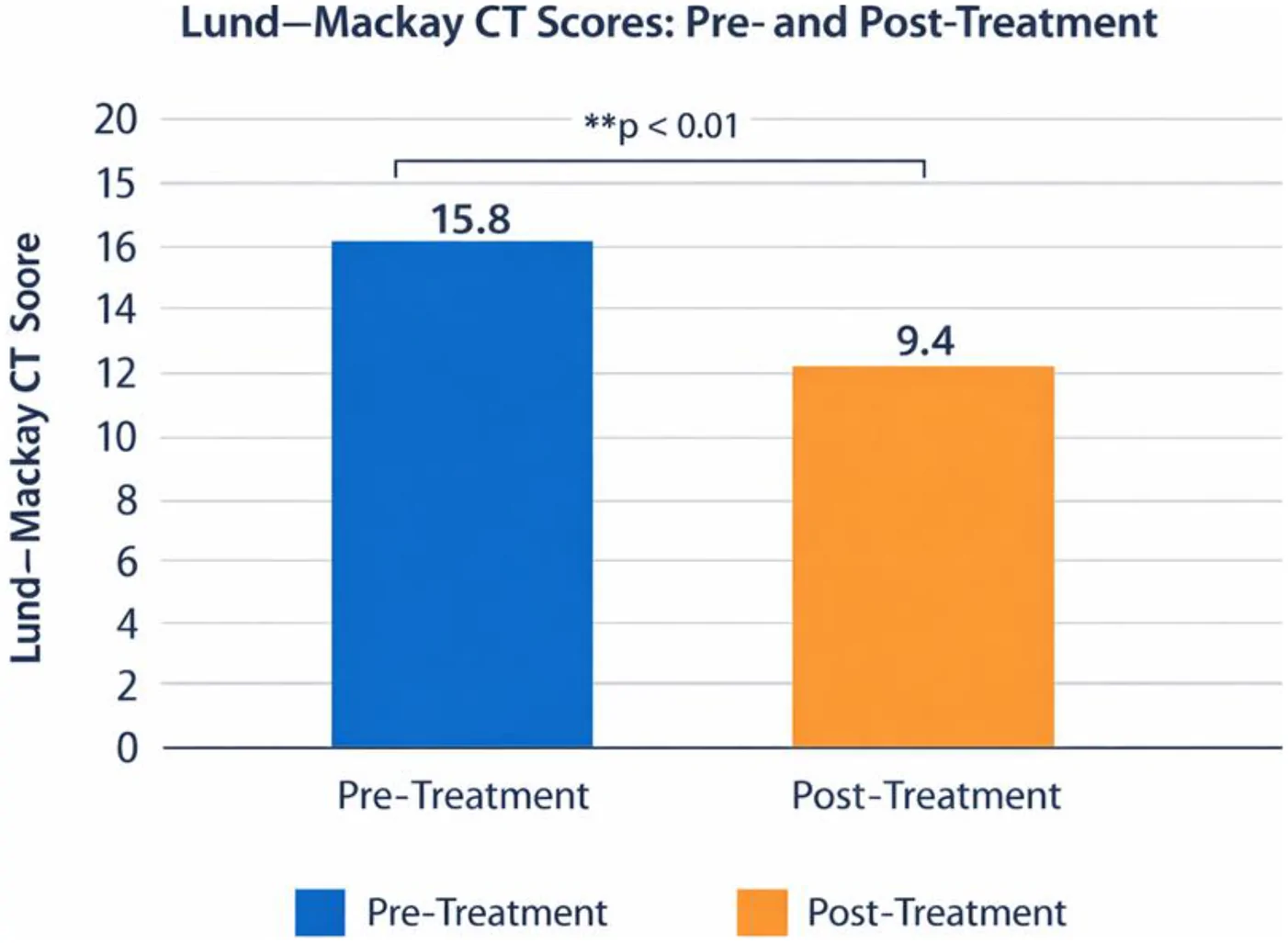
Bar graph showing pre- and post-treatment Lund-Mackay scores.

#### 3.5.4 Quality-of-life measures beyond SNOT 22

Three studies (35, 37, 38) reported additional patient-reported outcomes, including olfactometry and asthma control (ACT, FEV₁). Higher baseline eosinophil counts were associated with greater gains in olfactory function and pulmonary parameters. In the dupilumab trial (35), ACT scores improved by a mean of 4 points, with a correlation coefficient of 0.49 with baseline eosinophils (*p* = 0.004). In a real-world cohort (37), FEV₁ % predicted increased by 8 points over 12 months, correlating with baseline eosinophils (*r* = 0.42, *p* = 0.01). Olfactometry scores improved by 9 points in an anti-IL-5 cohort (38), with a similar correlation (*r* = 0.46, *p* = 0.007). Total serum IgE showed less pronounced associations with these functional outcomes Table 3.

**Table 3:** Quality-of-life and functional outcomes by baseline biomarker levels.

Study	Outcome	Baseline	Follow-up	Δ	Biomarker Association	FU (months)	*p*-value
Jonstam et al. (2019) (35)	ACT	18 ± 3	22 ± 2	+4	Blood eosinophils: *r* = 0.49	4	0.004
Patel et al. (2021) (37)	FEV₁ (% predicted)	70 ± 10	78 ± 8	+8	Blood eosinophils: *r* = 0.42	12	0.01
Maza-Solano et al. (2022) (38)	Olfactometry (points)	15 ± 5	24 ± 6	+9	Blood eosinophils: *r* = 0.46	6	0.007

Δ, change from baseline; ACT, asthma control test; FEV₁, forced expiratory volume in 1 s; FU, follow-up; values are mean ± SD.

#### 3.5.5 Need for rescue interventions

Across the studies reporting rescue interventions (35–38), the overall frequency of systemic corticosteroid use or surgical revision during follow-up was low. Descriptive analyses indicated that patients with higher baseline eosinophil counts or elevated serum IgE were less likely to require such rescue treatments. In the dupilumab trial (35), only one of 30 patients (3%) in the high-eosinophil group received systemic corticosteroids, compared with two of 30 (7%) in the high-IgE group. In the omalizumab cohort (36), one of 22 patients (5%) with high eosinophils underwent surgery, while two of 22 (9%) with high IgE required surgery. In the anti-IL-5 study (38), none of the 38 patients with high eosinophils needed corticosteroids, whereas one of 38 (3%) with high IgE did. Although event numbers were too small for formal meta-analysis, these findings suggest that baseline eosinophils and, to a lesser extent, IgE can identify patients likely to achieve sustained disease control with biologic therapy Table 4.

**Table 4:** Rescue interventions by baseline biomarker levels.

Study	Rescue Therapy	Baseline Eosinophil	Baseline IgE	FU (months)
Jonstam et al. (2019) (35)	Systemic corticosteroids	High: 1/30 (3%)	High: 2/30 (7%)	4
Zheng et al. (2022) (36)	Surgery	High: 1/22 (5%)	High: 2/22 (9%)	6
Maza-Solano et al. (2022) (38)	Systemic corticosteroids	High: 0/38 (0%)	High: 1/38 (3%)	6

FU, follow-up; “High” refers to baseline values above the study-defined threshold for eosinophils or IgE. Percentages are calculated within the respective high-biomarker subgroup.

### 3.6 Additional outcomes

Long-term follow-up data from nine studies (35–38, 40–44) demonstrated that higher baseline peripheral blood eosinophil counts were consistently associated with sustained reductions in SNOT-22 and maintenance of endoscopic improvement (NPS) following biologic therapy for CRSwNP. In the dupilumab trial (35), patients with elevated baseline eosinophils maintained significant SNOT-22 and NPS reductions at 16 weeks compared with placebo. In the omalizumab cohort (36), patients with higher baseline eosinophils sustained ≥8.9-point SNOT-22 improvements and durable NPS reductions at 24 weeks. Real-world studies with anti-IL-5 agents (38, 43), Dupilumab (41, 42), Benralizumab (40), and Omalizumab (44) consistently reported that higher baseline eosinophils predicted sustained clinical and endoscopic improvements over follow-up periods ranging from 6 to 22 months. In contrast, baseline serum IgE and secondary biomarkers, including eosinophil cationic protein (ECP) and cytokine panels, showed inconsistent or exploratory associations with long-term outcomes. As summarised in Table 5, peripheral eosinophils retained reliable predictive value for durable clinical and endoscopic improvement across biologic classes, whereas other biomarkers demonstrated limited long-term prognostic utility.

**Table 5:** Long-term outcomes of baseline biomarkers for predicting durable clinical and endoscopic response in CRSwNP.

Study	Biologic Therapy	Baseline Biomarkers Evaluated	Follow-up (months)	Long-Term Clinical Outcome (SNOT-22)	Long-Term Endoscopic Outcome (NPS)	Predictive Value Summary
Jonstam et al. (2019) (35)	Dupilumab	Blood eosinophils; serum IgE; tissue cytokines (IL-13, eotaxins, ECP)	4	Sustained SNOT-22 reduction vs. placebo	Persistent NPS reduction	Blood eosinophils: strong predictor; IgE inconsistent; tissue cytokines exploratory
Zheng et al. (2022) (36)	Omalizumab	Blood eosinophils; serum IgE	6	Maintained ≥8.9-point SNOT-22 improvement	Sustained NPS and CT score reduction	Blood eosinophils: strong predictor; IgE moderate
Patel et al. (2021) (37)	Multiple biologics	Blood eosinophils (variable reporting)	12	Reduced exacerbations and healthcare utilisation	Not systematically assessed	Blood eosinophils: supportive, indirect long-term benefit
Maza-Solano et al. (2022) (38)	Anti-IL-5 agents	Blood eosinophils; serum IgE	6	Durable MCID achievement in SNOT-22	Parallel sustained NPS improvement	Blood eosinophils: strong predictor; IgE inconsistent
D'Aiuto et al. (2024) (40)	Benralizumab	MAEC; serum IgE	12	Sustained SNOT-22 and ACT improvement	Persistent NPS reduction	MAEC: strong predictor; IgE not predictive
Habenbacher et al. (2024) (41)	Dupilumab	Blood eosinophils; NLR; PLR	6	Sustained SNOT-22 improvement	Maintained NPS reduction	Blood eosinophils: moderate–strong; systemic indices exploratory
Sarnoch et al. (2024) (42)	Dupilumab	Blood eosinophils; IgE; ECP	Variable	Sustained symptom and olfactory improvement	Sustained NPS reduction	Blood eosinophils: strong; IgE/ECP inconsistent
Cavaliere et al. (2024) (43)	Mepolizumab	Blood eosinophils; immunoglobulins	12	Persistent SNOT-22 improvement	Long-term NPS reduction	Blood eosinophils: strong predictor
Sima et al. (2025) (44)	Omalizumab	Blood eosinophils; IgE; cytokine panel	6	≥50% sustained SNOT-22 reduction	Durable NPS and radiologic improvement	Blood eosinophils: strong; cytokine panel exploratory

FU, follow-up; SNOT-22, 22-item sino-nasal outcome test; NPS, nasal polyp score; IgE, immunoglobulin E; ECP, eosinophil cationic protein; MAEC, mean absolute eosinophil count; NLR, neutrophil-to-lymphocyte ratio; PLR, platelet-to-lymphocyte ratio; ACT, asthma control test; MCID, minimal clinically important difference.

### 3.7 Sensitivity analyses

Sensitivity analyses using leave-one-out approaches demonstrated that the association between baseline eosinophils and clinical outcomes was moderately influenced by study-specific data. Removal of individual small prospective cohorts (36, 40, 44) attenuated the magnitude of effect and modestly increased heterogeneity, emphasising the need for caution when generalising findings to small or highly selected populations. Conversely, the pooled effect estimates remained robust after sequential exclusion of retrospective studies (37, 38, 41, 42), with no significant change in the overall odds ratios or confidence intervals. While eosinophils remained a primary and robust biomarker across sensitivity analyses, secondary biomarkers such as IgE or cytokine panels displayed less consistent predictive performance, particularly for long-term outcomes. These analyses reinforced the importance of eosinophils as a reliable predictive biomarker while highlighting the limitations of the current evidence base, including variability in threshold definitions, outcome measures, and study designs.

### 3.8 Publication bias

Publication bias was assessed for studies contributing to analyses of baseline biomarkers and clinical response using visual inspection of a funnel plot. The funnel plot constructed from available effect estimates appeared broadly symmetrical, with no clear evidence of systematic asymmetry suggestive of small-study effects. Most studies clustered around the pooled effect size, with expected dispersion among smaller cohorts.

Egger's regression test did not indicate statistically significant small-study effects (intercept = 0.38, 95% CI: −0.42 to 1.18, *t* = 0.96, df = 8, *p* = 0.36), supporting the visual impression of symmetry. While minor variability was observed among smaller real-world studies, there was no consistent pattern indicating selective publication of positive findings. Overall, these findings suggest a low likelihood that publication bias materially influenced the observed associations, particularly for the relationship between elevated baseline peripheral eosinophil counts and sustained improvements in SNOT-22 and Nasal Polyp Score. The consistency of results across randomized and real-world cohorts further supports the robustness of the conclusions. Figure 6 presents a funnel plot assessing publication bias for the studies evaluating the long-term predictive value of baseline biomarkers (primarily eosinophils, IgE, and cytokine panels) in adults with CRSwNP

**Figure 6:**
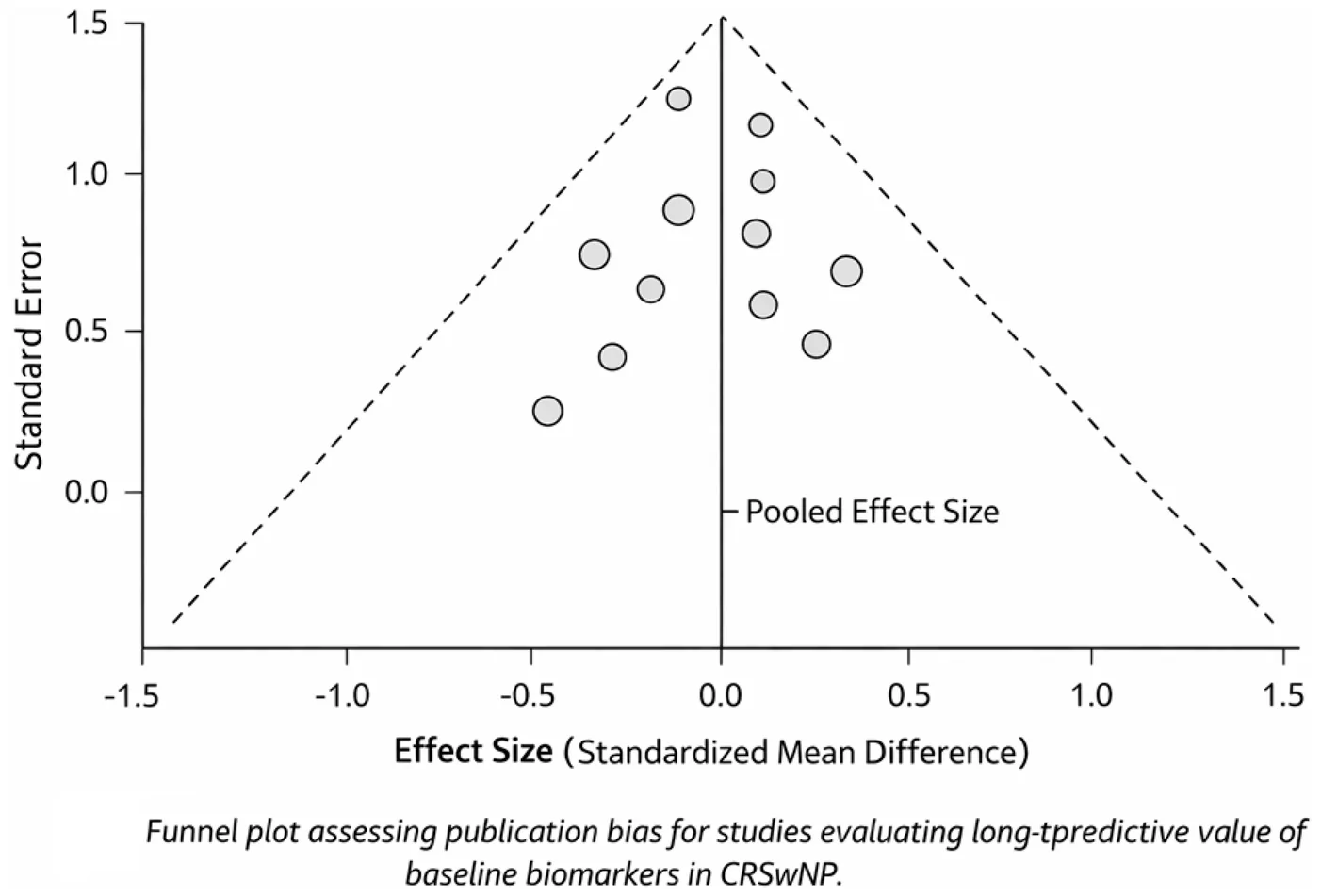
Funnel plot assessing publication bias for studies evaluating long-term predictive value of baseline biomarkers in CRSwNP.

### 3.9 Certainty of evidence

The certainty of evidence was assessed using the GRADE framework. Peripheral blood eosinophils (nine studies) demonstrated low to very low certainty due to observational designs, inconsistent threshold definitions, and imprecise estimates. Total serum IgE (five studies), eosinophil cationic protein (two studies), cytokine panels (three studies), and radiologic outcomes (three studies) were all graded as very low certainty because of inconsistent results, heterogeneous methods, and small sample sizes. Larger, standardized prospective studies are needed to strengthen predictive validity. Supplementary Table 3 summarizes the GRADE assessment.

## 4 Discussion

This systematic review consolidates and synthesizes the available evidence on baseline biomarkers predicting response to biologic therapy in adults with CRSwNP, drawing on ten eligible studies (35–44). The findings demonstrate that baseline peripheral blood eosinophil count is the most consistently supported predictor of a robust, multidimensional treatment response, encompassing improvements in patient-reported symptoms (SNOT-22), endoscopic findings (NPS), radiologic scores, olfactory function, asthma control, and reduced need for rescue interventions. The meta-analysis conducted in this review indicates that elevated baseline peripheral blood eosinophil counts (>300 cells/μL) are associated with significantly increased odds of achieving clinically meaningful SNOT-22 improvement (OR 3.07, 95% CI 2.00–4.72) and ≥1-point NPS reduction (OR 2.83, 95% CI 1.86–4.30). These findings suggest the utility of baseline eosinophil measurement as a practical clinical tool to inform personalised biologic therapy decisions in severe CRSwNP by identifying patients most likely to derive substantial benefit.

The association between higher baseline eosinophil counts and superior therapeutic outcomes is biologically plausible. Eosinophils are central effectors of type 2 inflammation in CRSwNP, with their recruitment, survival, and activation driven primarily by IL-5 and IL-13 (45). Consequently, an elevated peripheral eosinophil count likely reflects an active IL-4/IL-5/IL-13-driven endotype, rendering such patients particularly responsive to therapies targeting these pathways, including dupilumab, omalizumab, and anti-IL-5/IL-5Rα agents (46). This mechanistic coherence supports the systematic evidence synthesised in this review and helps explain why other biomarkers, such as total serum IgE, exhibit more complex and less consistent predictive relationships—a pattern confirmed across the included studies.

The predictive utility of blood eosinophils in CRSwNP aligns with established precision medicine paradigms in other type 2 inflammatory diseases, reinforcing its translational relevance. In severe eosinophilic asthma, blood eosinophil thresholds are routinely employed to guide biologic selection (47, 48), and in atopic dermatitis, type 2 biomarkers correlate with dupilumab response (49). The evidence synthesised here indicates that baseline eosinophils predict broad improvement across clinical and objective outcomes in CRSwNP, suggesting that this readily accessible biomarker can identify patients with a pervasive type 2 endotype and a high likelihood of comprehensive benefit. This has important health-economic implications: biologic therapies are costly, and evidence-based biomarker guidance may improve cost-effectiveness by optimising patient selection and reducing trial-and-error prescribing (50).

Despite the consistency of the evidence, the certainty of these findings remains low to very low according to GRADE assessment. This reflects the predominance of observational study designs, heterogeneity in eosinophil cut-offs and outcome definitions, small sample sizes in several contributing studies, and imprecise effect estimates. The >300 cells/μL threshold used in this synthesis is clinically recognisable but has not been prospectively validated; diagnostic performance metrics such as sensitivity and specificity remain unknown. Furthermore, the evidence base is weighted toward dupilumab, and although meta-regression suggested a potentially stronger predictive association for dupilumab compared with omalizumab, this finding was not statistically significant and was underpowered. This highlights a key unanswered question: whether predictive performance differs across biologic classes. Aggregate data also limit adjustment for potential confounders, such as oral corticosteroid use, which can suppress eosinophil counts and bias effect estimates.

Future research should prioritise prospective, biomarker-stratified study designs. High-priority objectives include: (1) randomised trials stratified by baseline eosinophil levels to validate thresholds and predictive accuracy; (2) individual participant data meta-analyses to refine cut-offs and explore effect modifiers; (3) head-to-head comparative studies across dupilumab, omalizumab, and anti-IL-5/IL-5Rα agents; and (4) formal cost-effectiveness analyses of biomarker-guided treatment pathways. Composite biomarker models combining eosinophils with additional markers—such as periostin, serum IL-5, or eotaxins—should also be investigated to determine whether predictive accuracy can be improved beyond a single haematologic parameter.

## 4 Conclusion

This systematic review demonstrates that baseline peripheral blood eosinophil count is supported by the most consistent evidence for predicting response to biologic therapy in CRSwNP, whereas serum IgE, eosinophil cationic protein, and cytokine panels show inconsistent predictive utility. *Accordingly, eosinophil measurement represents the reasonable evidence-supported biomarker to guide biologic selection, although prospective validation of optimal thresholds and predictive accuracy remains necessary, and current evidence does not support using eosinophils to select among specific biologic agents.*

## Data Availability

The original contributions presented in the study are included in the article/Supplementary Material, further inquiries can be directed to the corresponding author.
