# Supplementary material for: Predictive biomarkers of biologic therapy response in chronic rhinosinusitis with nasal polyps: a systematic review
Source: Front Med (Lausanne). 2026 Sep 16;13:1793335. doi: 10.3389/fmed.2026.1793335 (PMC13628775; doi:10.3389/fmed.2026.1793335)
Supplement: Supplementary file 1 [file Table1.docx]

### **Supplementary Table 1. Complete Search Syntax for Electronic Databases**

| **Database** | **Search String (Executed on January 2025)** | **Notes** |
| --- | --- | --- |
| **PubMed** | ("Rhinosinusitis"[Mesh] OR "Chronic Disease"[Mesh] OR "Sinusitis"[Mesh] OR "chronic rhinosinusitis"[tiab] OR CRS[tiab] OR "chronic sinusitis"[tiab]) AND ("Nasal Polyps"[Mesh] OR "nasal polyp*"[tiab] OR CRSwNP[tiab]) AND ("Biological Therapy"[Mesh] OR "Antibodies, Monoclonal"[Mesh] OR "Antibodies, Monoclonal, Humanized"[Mesh] OR "Molecular Targeted Therapy"[Mesh] OR biologic*[tiab] OR dupilumab[tiab] OR omalizumab[tiab] OR mepolizumab[tiab] OR benralizumab[tiab] OR reslizumab[tiab]) AND ("Biomarkers"[Mesh] OR biomarker*[tiab] OR predict*[tiab] OR "prognostic" OR "Treatment Outcome"[Mesh] OR response[tiab] OR responder*[tiab] OR "eosinophil*"[tiab] OR "Immunoglobulin E"[Mesh] OR IgE[tiab] OR "cytokine*"[tiab]) | Utilizes MeSH terms and free-text terms. Filters: Humans, Adult ≥19 years. Date range: inception to January 2025. |
| **Embase (via Ovid)** | 1. exp chronic rhinosinusitis/ or (chronic rhinosinusitis or CRS).ti,ab,kw. 2. exp nasal polyp/ or (nasal polyp* or CRSwNP).ti,ab,kw. 3. 1 and 2 4. exp biological therapy/ or exp monoclonal antibody/ or (biologic* or dupilumab or omalizumab or mepolizumab or benralizumab or reslizumab).ti,ab,kw. 5. exp biomarker/ or (biomarker* or predict* or prognostic or response or responder*).ti,ab,kw. 6. 3 and 4 and 5 7. limit 6 to (human and adult) | Uses Emtree thesaurus terms (exp) and free-text searches. Limits applied as final step. Date range: inception to January 2025. |
| **Scopus** | TITLE-ABS-KEY ( ( "chronic rhinosinusitis" OR CRS OR "chronic sinusitis" ) AND ( "nasal polyp*" OR CRSwNP ) AND ( biologic* OR dupilumab OR omalizumab OR mepolizumab OR benralizumab OR reslizumab OR "monoclonal antibody" ) AND ( biomarker* OR predict* OR prognostic OR response OR responder* OR eosinophil* OR IgE OR cytokine* ) ) AND ( LIMIT-TO ( DOCTYPE , "ar" ) OR LIMIT-TO ( DOCTYPE , "re" ) ) AND ( LIMIT-TO ( SRCTYPE , "j" ) ) | Searches title, abstract, and keywords. Limits to journal articles and reviews. Date range: inception to January 2025. |
| **Web of Science Core Collection** | TS=((("chronic rhinosinusitis" OR "CRS" OR "chronic sinusitis") AND ("nasal polyp*" OR "CRSwNP") AND (biologic* OR dupilumab OR omalizumab OR mepolizumab OR benralizumab OR reslizumab) AND (biomarker* OR predict* OR prognostic OR response OR responder* OR eosinophil* OR IgE OR cytokine*))) Refined by: Document Types: (ARTICLE OR REVIEW), Web of Science Categories: (ALLERGOLOGY OR OTORHINOLARYNGOLOGY OR IMMUNOLOGY OR MEDICINE GENERAL INTERNAL) | TS searches the Topic field (title, abstract, author keywords, Keywords Plus®). Category filters increase clinical relevance. Date range: inception to January 2025. |
| **Cochrane CENTRAL (via Wiley)** | #1 MeSH descriptor: [Rhinosinusitis] this term only #2 MeSH descriptor: [Nasal Polyps] this term only #3 ("chronic rhinosinusitis" or CRS):ti,ab,kw #4 ("nasal polyp*" or CRSwNP):ti,ab,kw #5 (#1 OR #3) AND (#2 OR #4) #6 MeSH descriptor: [Biological Therapy] this term only #7 MeSH descriptor: [Antibodies, Monoclonal] this term only #8 (biologic* or dupilumab or omalizumab or mepolizumab or benralizumab or reslizumab):ti,ab,kw #9 #6 OR #7 OR #8 #10 MeSH descriptor: [Biomarkers] this term only #11 (biomarker* or predict* or response):ti,ab,kw #12 #10 OR #11 #13 #5 AND #9 AND #12 Publication Year from 1900 to 2025 | Uses Cochrane's PICO-focused search syntax. CENTRAL primarily contains trial records. Date range: inception to January 2025. |

**CRS: Chronic Rhinosinusitis, CRSwNP: Chronic Rhinosinusitis with Nasal Polyps, tiab: Title/Abstract (field code for PubMed),MeSH: Medical Subject Headings, exp: Explode (thesaurus term command in Embase/Ovid), kw: Keyword (field code for Embase), TS: Topic (field code for Web of Science), PMID: PubMed Unique Identifier, CENTRAL: Cochrane Central Register of Controlled Trials**
