# Supplementary material for: Predictive biomarkers of biologic therapy response in chronic rhinosinusitis with nasal polyps: a systematic review
Source: Front Med (Lausanne). 2026 Sep 16;13:1793335. doi: 10.3389/fmed.2026.1793335 (PMC13628775; doi:10.3389/fmed.2026.1793335)
Supplement: Supplementary file 2 [file Table2.docx]

**Supplementary Table 2: Newcastle-Ottawa Scale (NOS) Assessment for Observational Studies [37–45]**

| Study | Selection (Max 4 ★) | Comparability (Max 2 ★) | Outcome (Max 3 ★) | Total Score (Max 9 ★) |
| --- | --- | --- | --- | --- |
| Zheng et al., 2022 [37] | ★★★★ | ★★ | ★★★ | 9 |
| Patel et al., 2021 [38] | ★★★ | ★★ | ★★★ | 8 |
| Maza-Solano et al., 2022 [39] | ★★★ | ★★ | ★★★ | 8 |
| De Corso et al., 2023 [40] | ★★★★ | ★★ | ★★★ | 9 |
| D'Aiuto et al., 2024 [41] | ★★★ | ★★ | ★★ | 7 |
| Habenbacher et al., 2024 [42] | ★★★ | ★★ | ★★★ | 8 |
| Sarnoch et al., 2024 [43] | ★★★ | ★★ | ★★★ | 8 |
| Cavaliere et al., 2024 [44] | ★★★★ | ★★ | ★★★ | 9 |
| Sima et al., 2025 [45] | ★★★★ | ★★ | ★★★ | 9 |

NOS, Newcastle-Ottawa Scale; Max, maximum; ★, star awarded for meeting quality criterion.
