# Supplementary material for: Predictive biomarkers of biologic therapy response in chronic rhinosinusitis with nasal polyps: a systematic review
Source: Front Med (Lausanne). 2026 Sep 16;13:1793335. doi: 10.3389/fmed.2026.1793335 (PMC13628775; doi:10.3389/fmed.2026.1793335)
Supplement: Supplementary file 3 [file Table3.docx]

**Supplementary Table 3. GRADE Assessment of Baseline Biomarkers for Predicting Response to Biologic Therapy in CRSwNP**

| Biomarker Category | Studies (n) | Limitations | Inconsistency | Indirectness | Imprecision | Publication Bias | Overall Certainty |
| --- | --- | --- | --- | --- | --- | --- | --- |
| Peripheral blood eosinophils | 9 [36–45] | Serious¹ | Serious² | Not serious | Serious³ | Undetected | ⨁◯◯◯ Very low |
| Total serum IgE | 5 [36–39,43] | Serious¹ | Serious⁴ | Not serious | Serious³ | Undetected | ⨁◯◯◯ Very low |
| Eosinophil cationic protein (ECP) | 2 [36,43] | Serious¹ | NA⁵ | Not serious | Very serious⁶ | Undetected | ⨁◯◯◯ Very low |
| Cytokine panels (IL‑5, eotaxins, periostin) | 3 [36,37,45] | Serious¹ | Serious⁷ | Serious⁸ | Serious³ | Undetected | ⨁◯◯◯ Very low |
| Radiologic outcomes (Lund‑Mackay CT) | 3 [36,37,45] | Serious¹ | Serious⁹ | Not serious | Serious³ | Undetected | ⨁◯◯◯ Very low |

ECP: eosinophil cationic protein; CT: computed tomography; NA: not applicable.

**Footnotes:**
¹ Observational design in most studies, variable biomarker measurement methods, and lack of standardised thresholds.
² Heterogeneous cut‑offs (300–500 cells/μL) and outcome definitions.
³ Moderate to wide confidence intervals; limited sample sizes.
⁴ Inconsistent predictive utility across studies.
⁵ Insufficient number of studies to assess consistency.
⁶ Very small sample sizes; wide confidence intervals.
⁷ Heterogeneous panels and analytical methods.
⁸ Exploratory analyses; no validation cohorts.
⁹ Variable scoring methods (Lund‑Mackay) and reporting.
